# A Chinese family with cat eye syndrome and abnormality of eye movement: First case report

**DOI:** 10.3389/fped.2023.1145183

**Published:** 2023-04-11

**Authors:** Yang Lu, Liping Shen, Yue Zheng, Haichen Zhang, Yanbo Liu, Ming Qi, Shangzhi Huang, Bo Shen

**Affiliations:** ^1^ Health Clinic Center for Enze Precision Medicine, Taizhou Hospital of Zhejiang Province Affiliated to Wenzhou Medical University, Taizhou, China; ^2^ Department of Ophthalmology, Taizhou Hospital of Zhejiang Province Affiliated to Wenzhou Medical University, Taizhou, China; ^3^ Department of Endocrinology, Peking Union Medical College Hospital, Beijing, China; ^4^ Clinical Genome Center, DIAN Diagnostics, Hangzhou, China; ^5^ Department of Pathology and Laboratory Medicine, University of Rochester, Rochester, NY, USA; ^6^ Department of Medical Genetics, Peking Union Medical College, Beijing, China

**Keywords:** cat eye syndrome (CES), 1.35 mb tetrasomy, abnormality of eye movement, first report, early diagnosis

## Abstract

**Background:**

Cat eye syndrome (CES) is a rare disease with a wide spectrum of phenotypic variability that is observed in 1:150,000 newborns. CES is characterized clinically by the combination of iris coloboma, anal atresia, and preauricular tags and/or pits. Many eye malformations have been reported to be associated with CES, such as iris and chorioretinal coloboma. However, an abnormality of eye movement has not been previously reported.

**Case presentation:**

We report on a Chinese family carrying a 22q11.1-q11.21 duplication of 1.7Mb tetrasomy (chr22:16,500,000–18,200,000, hg38) in two generations. Based on the proband and her father’s clinical manifestations, including ophthalmological examination, cytogenetic analysis, FISH, CNV-seq, and WES, the diagnosis of CES with an abnormality of eye movement was made.

**Conclusion:**

Our findings broadened the symptom spectrum of CES syndrome and laid the foundation for pathogenesis, diagnostic targets, and drug research on the abnormality of eye movement, and were helpful for early diagnosis and intervention of CES.

## Background

Cat eye syndrome (CES) (MIM #115470), also known as Schmid-Fraccaro syndrome, is mainly caused by chromosome 22q11 inversion duplication or chromosome 22 partial tetrasomy (1) and is observed in 1:150,000 newborns. The major clinical features of CES are the combination of anal atresia, preauricular pits and tags, and/or iris coloboma (2). In addition, most patients also present additional malformations involving the cardiovascular and nervous systems, indicating a very wide clinical spectrum in terms of features and severity (3). Chromosome 22q11 is an ideal substrate for non-allelic homologous recombination (NAHR) and is highly susceptible to chromosomal rearrangements (4). Therefore, the 22q11 region is associated with many microduplication and microdeletion conditions such as CES, chromosome 22q11.2 microduplication syndrome (dup22q11) (MIM #608363), velocardiofacial syndrome (VCFS) (MIM #192430), and DiGeorge syndrome (DGS) (MIM #188400) (5, 6). Due to reciprocal rearrangements, 22q11.2 duplications and deletions are predicted to occur at the same frequency. However, the number of duplication cases, such as CES and dup22q11, reported are considerably less, which is likely because the duplications have a milder clinical effect. Affected dup22q11 individuals may have developmental delay, short stature, and facial abnormalities (7). The phenotypes of this condition like CES are highly variable, even among members of the same family. The chromosomal location and phenotype of CES are similar to those of dup22q11, which requires systematic differential diagnosis. Many eye malformations have been reported to be associated with CES, such as iris and chorioretinal coloboma, hypertelorism, and epicanthus. However, abnormality of eye movement is associated with Duane retraction syndrome (DURS) (MIM %126800, #604356 and #617041), congenital fibrosis of extraocular muscles (CFEOM) (MIM #135700, #602078, #600638, %609384, #616219), and abnormality of the optic nerve, as previously reported, and has not been associated with CES.

In the present study, we report on a Chinese family carrying a 22q11.1-q11.21 duplication of 1.7Mb tetrasomy (chr22:16,500,000–18,200,000, hg38) in two generations. The proband and her father presented with an abnormality of eye movement and were diagnosed with CES by ophthalmological examination, karyotyping, CNV-seq, and FISH. WES did not detect gene mutations associated with DURS, CFEOM, or abnormality of the optic nerve in the proband or father. Our findings broadened the symptom spectrum of CES syndrome, and abnormality of eye movement was noted in 22q11 for the first time. This laid the foundation for the pathogenesis, diagnostic targets, and drug research on the abnormality of eye movement.

### Case presentation

**Figure 1:**
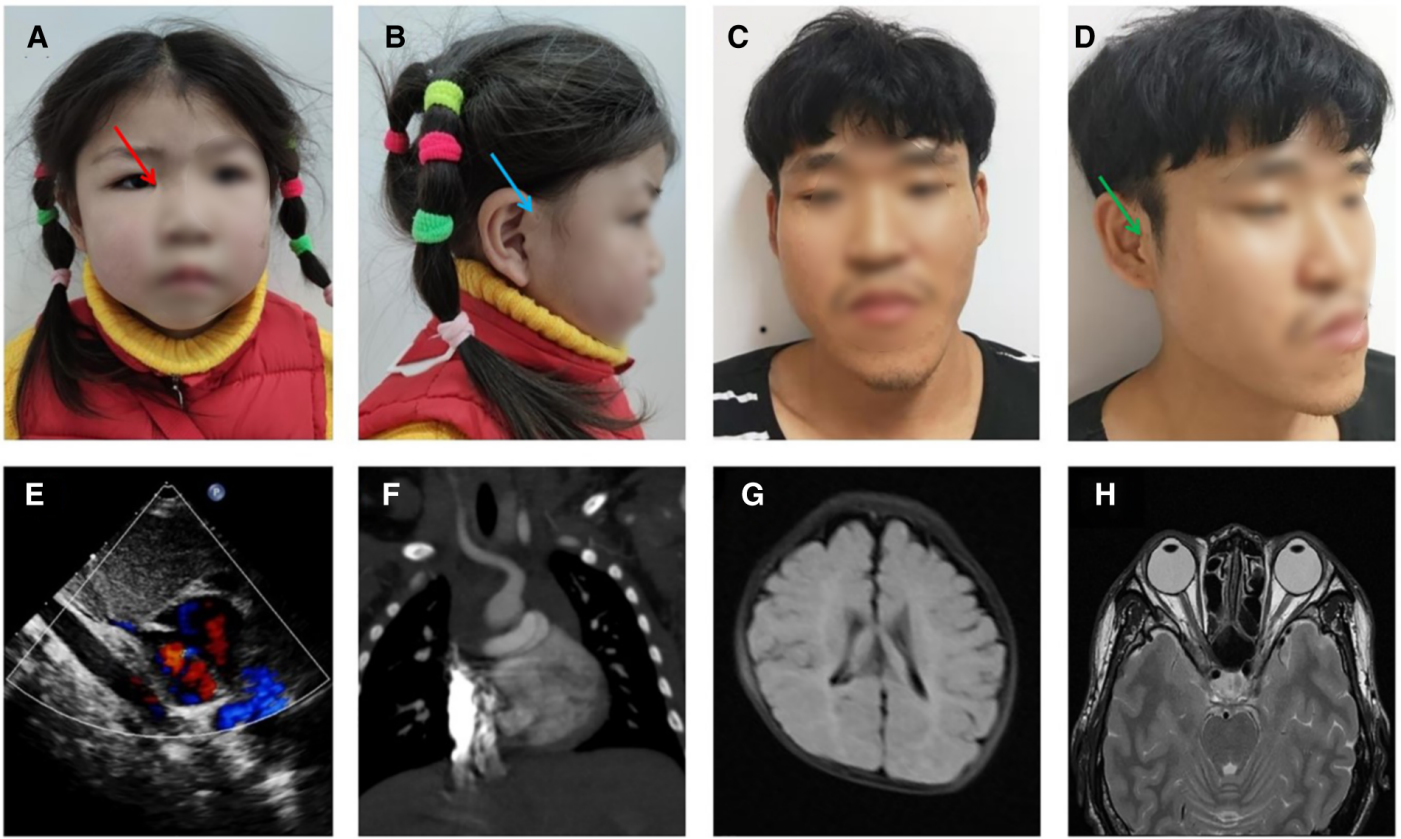
Craniofacial features and imaging results of the proband and father. (**A,B**) photograph of the proband at 4 years and 5 months: facial asymmetry, epicanthus (red arrow), and preauricular pits (blue arrow), (**C,D**) photograph of the father: facial asymmetry, epicanthus, and preauricular pits and kin tags (green arrow), (**E**) heart ultrasound of proband (1 M), (**F**) pulmonary artery CTA of the proband (4 M), (**G**) head MRI of the proband (4 M), (**H**) head MRI of the father.

The proband (III-1) is a girl aged 4 years and 5 months with congenital anal atresia, rectoperineal fistula, and chorioretinal coloboma. She came from a village located in the Zhejiang Province, China, and was the first child of a non-consanguineous couple. She was born to a 33-year-old mother G1P1 at 39 + 4 weeks by cesarean section due to hydramnios. Her birth weight was 2,850 g (19.3th percentile), birth length was 50.0 cm (67.5th percentile), head circumference was 34.0 cm (54.1th percentile), and Apgar scores were 9 at 1 min, 10 at 5 min, and 10 at 10 min. She was in the hospital with cyanosis at 1 month old, and echocardiography showed patent foramen ovale (Figure 1E). Since 4 months of age, she was repeatedly in the hospital with pneumonia, and computed tomography angiography (CTA) showed pulmonary artery sling (Figure 1F). In addition, magnetic resonance imaging (MRI) showed delayed myelination (Figure 1G). The proband had anoplasty at 6 months old, and recovered well. She sat at 9 months old and walked at 19 months old. At the age of 53 months, based on the proband’s performance on the Peabody Developmental Motor Scales (PDMS-II) and Gesell Development Scale (GDS), she had inadequate overall motor abilities and mild global developmental delay (for test results, see Supplementary Tables S1, S2). The difference between these two scores was not statistically significant, indicating that her fine and gross motor skills appeared to be approximately the same. Physical examination showed that her height was 98.1 cm (2.7th percentile), her weight was 15.1 kg (18.2th percentile) and her head circumference was 49.1 cm (35.1th percentile). She was found to have dysmorphic features, including short stature, moderate sensorineural hearing impairment, hypertelorism (Figure 1A), hypotonia, and preauricular pits (Figure 1B, blue arrow). Additional features included epicanthus (Figure 1A, red arrow), mild facial asymmetry (Figure 1A), and bilateral single transverse palmar creases.

The proband’s father (II-1) is 35 years old and had a low BMI, hypertelorism (Figure 1C), anal atresia, preauricular pits (Figure 1D), and skin tags (Figure 1D, green arrow). Additional features included epicanthus (Figure 1C), upslanted palpebral fissure (Figure 1D), severe facial asymmetry (Figure 1C), scoliosis, mild intellectual disability, and nasal speech.

### Ophthalmological examination results

**Figure 2:**
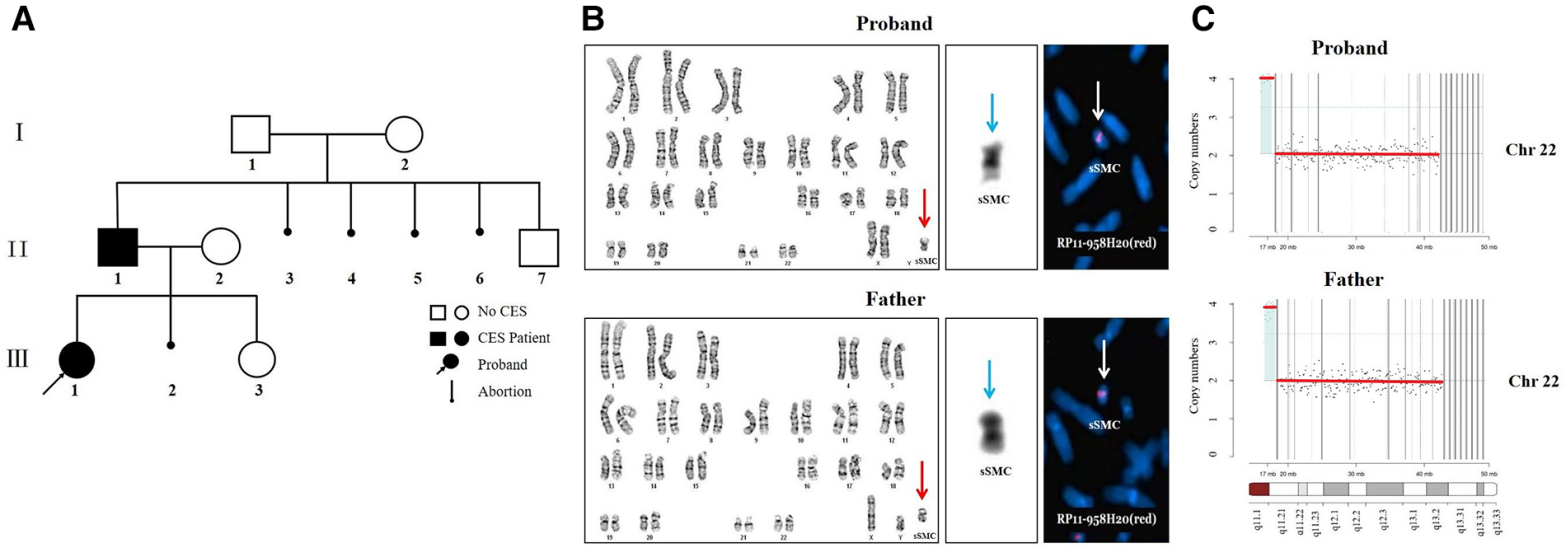
(**A**) the family pedigree. The basic information of family members is as follows. I-1: 55 years old, normal phenotype with a normal male karyotype. I-2: 54 years old, moderate intellectual disability with a normal female karyotype, mild short stature, hypertelorism, no facial asymmetry, no abnormality of eye movement, no anal atresia, and no preauricular pits or skin tags. II-2: 38 years old, mild intellectual disability with a normal female karyotype, low-set ears, hypertelorism, no facial asymmetry, no abnormality of eye movement, no anal atresia, no preauricular pits, and no teratogenic drugs during pregnancy, 22q11.21 (18,340,000–21,100,000, hg38) x3. II-3, II-4, II-5, II-6 elective artificial abortion. II-7, 21 years old, normal phenotype with a normal male karyotype. III-2 spontaneous abortion and no genetic testing. III-3, 8 months old, global developmental delay with a normal female karyotype, low-set ears, hypertelorism, preauricular pits, patent foramen ovale, no facial asymmetry, no abnormality of eye movement, and no anal atresia, 22q11.21 (18,340,000–21,100,000, hg38) x3. (**B**) The results of cytogenetic analysis and FISH. G-banding karyotype analyses of the proband and father showed sSMC (red arrow). C-banding karyotype analyses of the proband and father showed two centromeres in the sSMC (blue arrow). The FISH results of the proband and father showed two signals in the marker metaphase chromosome (white arrow). (**C**) The CNV-seq results of the proband and father.

**Figure 3:**
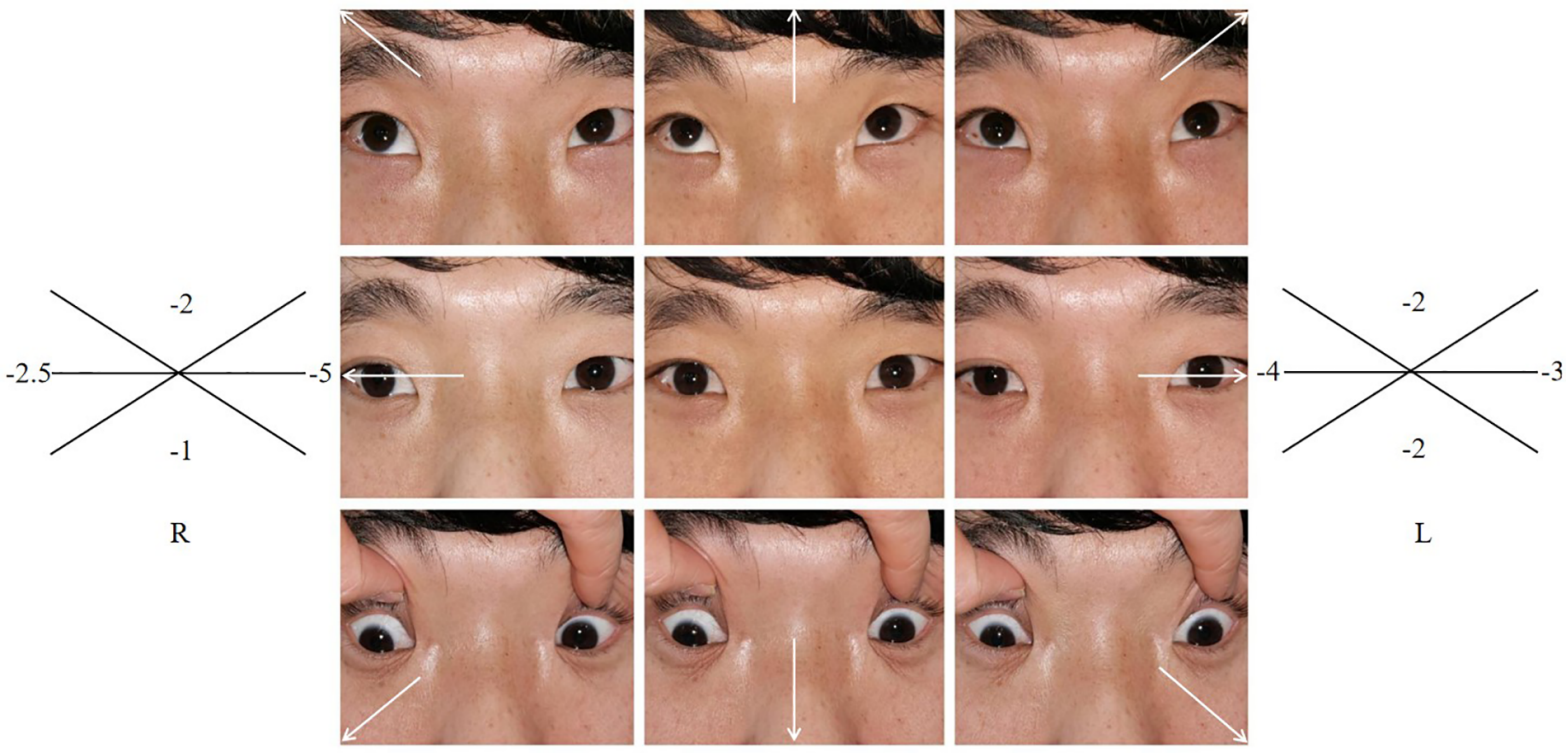
Eyeballs movement.

In this family (for family pedigree see Figure 2A), in addition to the typical symptoms of CES, the abnormality of eye movement was present. Comprehensive ophthalmic examination, such as refraction, intraocular pressure, anterior and posterior eyeball examination, visual field examination, eyeball movement examination, and the Krimsky test were used to assess deviation, showing that the proband and her father had astigmatism combined with mild myopia and limitation of ocular motility. There were no obvious palpebral fissure changes when their eyes moved. The proband’s father had 10^△^ exotropia in the right eye at the primary position and eye movement with limitations in all directions (Figure 3). Head magnetic resonance imaging (MRI) ruled out tumors, infection, and muscle edema in the proband’s father (Figure 1H). However, the proband refused examinations such as the eyeball pull test and head MRI. This finding has not been previously described in the phenotype of CES.

### Genetic findings

In search of the cause of multiple organ abnormalities, peripheral blood cultures from all family members were stimulated with standard methods, and chromosomes were analyzed by G-banding and C-banding. Karyotype analyses showed a small supernumerary marker chromosome (sSMC) in the proband’s and father’s peripheral blood lymphocyte cells (Figure 2B, red arrow). Their karyotypes were 47,XX,+mar and 47,XY,+mar, respectively. The C-banding karyotype analyses showed that there were two centromeres on the sSMC (Figure 2B, blue arrow). The FISH results of the proband’s and the father’s metaphase cells showed two signals on the sSMC, ish mar (RP11-958H20++ or enh) (Figure 2B, white arrow). We examined the genomic DNA of the proband and father by low-coverage massively parallel CNV-seq, according to the manufacturer’s protocols. CNV-seq identified a partial repeat of this 22q11 region with a size of approximately 1.7Mb on 22q11.1-q11.21 (16,500,000–18,200,000, hg38) (Figure 2C). The triplosensitivity (TS) score of the ClinGen database was 3, that is, there was sufficient evidence that the duplication of one copy can cause disease. The triple TS disease is CES. In addition, the proband and father did not have gene mutations associated with DURS (*CHN1*, *MAFB*, *SALL4,* etc.), CFEOM (*COL25A1*, *KIF21A*, *PHOX2A*, *TUBA1A*, *TUBB2B*, *TUBB3,* etc.), or abnormality of the optic nerve by WES.

## Discussion and conclusions

**Figure 4:**
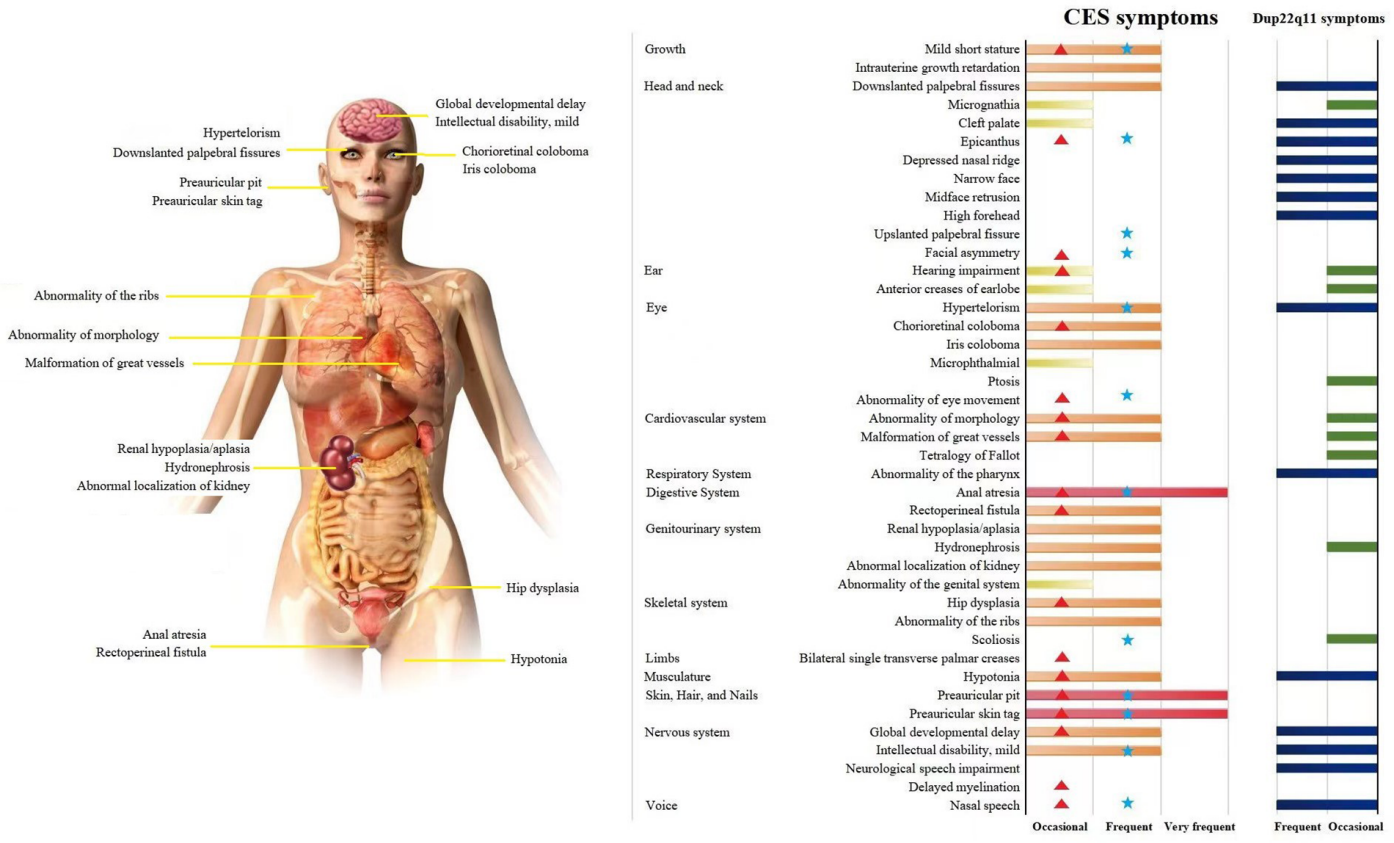
CES affects multiple systems and organs. A Comparison of CES and dup22q11 symptoms in the proband and father. Note: Symptoms and data source: OMIM, HPO, Orphanet, and 131 papers on CES published between 2001 and 2021. The frequency in the patient population: 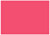, Very frequent (99%–80%); 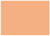 and 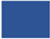, Frequent (79%–30%); 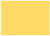 and 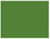, Occasional (29%–5%); 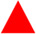, proband symptoms; 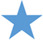, father symptoms.

CES covers a very wide spectrum of variable penetrance and expressivity, ranging from a normal phenotype to severe multisystemic symptoms. The three main characteristics are anal atresia, preauricular anomalies, and iris coloboma, but these characteristics are not consistently found in many patients with CES, as in our patients (2) (CES symptoms, Figure 4). The similarity of localization and symptoms makes the differential diagnosis between CES and dup22q11.2 difficult. A comparison of CES and dup22q11 symptoms with those of the proband and father showed that they suffered two very frequent phenotypes of CES (anal atresia and preauricular pits) and had a higher phenotype matching rate with CES than with dup22q11 (Supplementary Table S3) (for dup22q11 symptoms see Figure 4). According to the clinical manifestations of the proband and her father, the diagnosis of CES was made based on cytogenetic analysis and FISH showing the presence of a small supernumerary dicentric marker chromosome derived from chromosome 22q11. The CNV-seq results indicated that repeated regions included cat eye syndrome chromosome region, candidate 1 (*CECR1*), and candidate 2 (*CECR2*) genes located in a proximal 22q11 region, which further confirmed the previous diagnosis results.

In several previous reports, researchers found that abnormality of eye movement is associated with DURS, CFEOM, and abnormality of the optic nerve (8,9). There have been no reported cases of CES with an abnormality of eye movement. DURS was originally speculated to be the cause of this clinical condition. DURS is a congenital eye movement disorder with a prevalence of approximately 0.1% in the general population (9). DURS results in the restriction or absence of abduction, adduction, or both, and narrowing of the palpebral fissure and retraction of the globe on attempted adduction (10). CFEOM is part of a larger group of congenital cranial dysinnervation disorders and primarily affects ocular motility (8). However, the proband and her father had limited eye movement in the horizontal and vertical directions and had no features of retrograde eyeballs or palpebral fissure narrowing in attempted adduction, which excluded the possibility of CES combined with DURS. In addition, WES did not detect gene mutations associated with DURS, CFEOM, or abnormality of the optic nerve in the proband or father. Therefore, we think that the phenotype of eye movement disorder in the proband and the father is very likely related to the 1.7Mb tetrasomy. The 1.7Mb tetrasomy of the proband and her father on 22q11.1-q11.21 (16,500,000–18,200,000, hg38), which contains 11 OMIM morbid genes, namely, *XKR3*, *IL17RA*, *CECR1* or *ADA2*, *CECR2*, *SLC25A18*, *ATP6V1E1*, *BID*, *MICAL3*, *MIR648*, *PE*X26, and *TUBA8* (partially) (Supplementary Figure S1A). The proband and her father appeared to have a novel clinical symptom of eyeballs that could barely move. Compared with previously reported cases of CES and reviewing the function of the abovementioned genes (see Supplementary Table S4), *BID* and *MICAL3* are most likely associated with this new symptom. *BID* encodes a cell death agonist and regulates apoptosis, and this gene plays a role in inducing premature cell death and can influence organ development or overall growth (11). *MICAL3*, a plexin signaling molecule expressed in motor neurons (12), may be directly associated with impaired ocular movement.

Although the karyotype of the proband’s grandparents was normal, the possibility of germline mosaicism could not be ruled out. In addition, non-homologous recombination also likely produced the proband’s father’s sSMC. Intra-genome duplicates greater than 1 kb in length and with at least 90% sequence similarity are termed low-copy repeats (LCRs) (4). When two homologous LCRs are less than 10 Mb apart from each other, they result in chromosomal or chromatid mislocations and unequal crossovers, resulting in the deletion or duplication of genomic segments between LCRs (13). Chromosome 22q11.2 harbors eight LCRs termed LCR22A-LCR22H, therefore, LCR22s are an ideal substrate for non-allelic homologous recombination (NAHR), resulting in rearrangements of 22q11.2 (Supplementary Figures S1B,C) (14). Normal chromosome 22 is most likely to generate a 1.7Mb tetrasomy on 22q11.1-q11.21 (16,500,000–18,200,000, hg38) by mismatch and NAHR. Before the formation of the father’s sperm, chromosomes replicate in spermatogonial cells, but the small marker chromosome does not. During the first meiosis of the primary spermatocyte and homologous chromosomal synapsis, the same two sequences of 1.7Mb in the small marker chromosome were paired to form the structure, as shown in the genetic model. Usually, only one of two centromeres is functional and is randomly pulled into a secondary spermatocyte by the spindle fiber. Therefore, the father had a 50% probability of transmitting the sSMC to the offspring (Supplementary Figure S1D).

In summary, CES exhibits a broad spectrum of clinical features, and many mildly affected patients probably remain undetected. Here, we report on a Chinese family carrying a 22q11.1-q11.21 duplication of 1.7Mb tetrasomy (16,500,000–18,200,000, hg38) in two generations. The proband and her father were diagnosed with CES by karyotype analyses, CNV-seq, and FISH, and presented an abnormality of eye movement, which was reported for the first time. No gene mutations associated with eye movement disorders, such as DURS and CFEOM, were detected in the peripheral blood of the proband and father by WES. Therefore, we think that the phenotype of eye movement disorder in the proband and her father is very likely related to *BID* and *MICAL3* in the 1.7Mb tetrasomy. Our findings broadened the symptom spectrum of CES syndrome, and, for the first time, mutations causing abnormality of eye movement were located at 22q11. This laid the foundation for the pathogenesis, diagnostic targets, and drug research in abnormality of eye movement and was helpful for the early diagnosis and intervention of CES.

## Data Availability

The sequencing data presented in the study are deposited in the NCBI (ncbi.nlm.nih.gov/), accession numbers: SAMN33773438, SAMN33773439, SAMN33773440, SAMN33773441.
