# Supplementary material for: A Chinese family with cat eye syndrome and abnormality of eye movement: First case report
Source: Front Pediatr. 2023 Apr 11;11:1145183. doi: 10.3389/fped.2023.1145183 (PMC10126520; doi:10.3389/fped.2023.1145183)
Supplement: Supplementary file 1 [file Table1.docx]

**Supplementary information**

**Materials and methods**

The study was conducted in accordance with the tenets of the Declaration of Helsinki on human subjects. This study and all protocols used conformed to the ethical guidelines, as reflected in an a priori approval by the Ethics Committee of Taizhou Hospital of Zhejiang Province affiliated to Wenzhou Medical University. Informed consent was obtained from each patient included in the study, and written informed consent was obtained from all subjects.

**Ophthalmologic examination**

Ophthalmic examination, such as refraction, intraocular pressure, anterior and posterior eyeball examination, visual field examination, eyeballs movement examination and the Krimsky test, were used to assess deviation. The degree of eye movement restriction was defined as the classification of the degree of hyperfunction and insufficiency of extraocular muscles[1-2].

**Cytogenetic analysis**

Karyotyping was performed on all family members. Blood samples were obtained under sterile conditions, and blood was cultured for 3 days. Chromosomes were detected by G-banded and C-banded karyotyping using standard methods, and at least 20 metaphase cells were used for analysis. Resolution for the G-banded chromosomes was >550 bands.

**Fluorescence in situ hybridization (FISH)**

FISH analysis with a specific probe (RP11-958H20) for each of the autosome and sex chromosomes was performed on metaphases and interphases following standard methods. The probe binding region is 22q11.1-q11.21 (17,853,653-18,040,246).

**Copy number variation sequencing (CNV-seq)**

Low-coverage massively parallel copy number variation sequencing (CNV-seq) was detected in proband and father. Deoxyribonucleic acid (DNA) was extracted from each patient’s blood sample using DNeasy Blood & Tissue Kit (Qiagen). DNA samples were then fragmented, and the ends were repaired and ligated with adapters using the [NEBNext Ultra II DNA Library Prep Kit](https://www.so.com/link?m=br03MdjcHm59JuSnRSh5O2rbByBysPzWLAZaqUHriXSklb+JkmU+xJP/LZFwInD6pVNYp5t5Uht76tkQZhvDFM9bh2pvPAUy44SMAj9XI3oK8A3+L+CnCDjbqXEvrQkM7UIuGof7+ITzkm8cslPYOpBI2rQBm+g2WaklNc28ybhgnfp5x1DePjaaVpsS5vujplBUTfG2+mxg=) by Illumina. After PCR amplification, final quantified libraries were processed on Illumina PE150.

**Data process**

Sequencing reads were aligned to GRCh38 human reference using bowtie2. The analysis of CNV was conducted using WisecondorX software according to the previous research [PMID: 24170809, 29493577]. The aligned reads were converted to .npz files (both test and reference samples). The reference database was established using 30 healthy human samples (15 male and 15 female samples). With the .npz files of reference samples, WisecondorX will precisely predict copy number alterations of the test samples.

**Supplementary Table 1.** Test results of Peabody Developmental Motor Scales II.

| Subtests | Raw Scores | Age Equivalents | Standard Scores | Percentile | Quotient Scores | Description of Performance |
| --- | --- | --- | --- | --- | --- | --- |
| Reflexes (Re) | N/A | N/A | N/A | N/A |  | N/A |
| Stationary (St) | 49 | 49 months | 9 | 37 |  | Average |
| Locomotion (Lo) | 138 | 36 months | 6 | 9 |  | Below Average |
| Object Manipulation (Ob) | 33 | 39 months | 7 | 16 |  | Below Average |
| Gross Motor Quotient (GMQ) |  |  | 22 | 13 | 83 | Below Average |
| Grasping (GR) | 47 | 45 months | 7 | 16 |  | Below Average |
| Visual-Motor integration (Vi) | 126 | 46 months | 8 | 25 |  | Average |
| Fine Motor Quotient (FMQ) |  |  | 15 | 16 | 85 | Below Average |
| Total Motor Quotient (TMQ) |  |  | 37 | 12 | 82 | Below Average |

**Supplementary Table 2.** Test results of Gesell Developmental Schedules.

| GDS fields | Developmental Age (DA) | Developmental Quotient (DQ) | Description of Performance |
| --- | --- | --- | --- |
| Gross motor | 42.0 months | 79 | Borderline development |
| Fine motor | 39.0 months | 73 | Mild developmental delay |
| Adaptive behavior | 39.0 months | 73 | Mild developmental delay |
| Language | 43.5 months | 82 | Borderline development |
| Personal-social behavior | 42.0 months | 79 | Borderline development |

**Supplementary Table 3.** Clinical symptoms and genetic test data of proband and father.

|  | | Frequency | Proband III-1 | Proband’s father II-1 |
| --- | --- | --- | --- | --- |
| Growth | Gender/Age |  | Female /4 years and 5 months | Male /35 years |
|  | Height |  | 98.1cm (2.7th percentile) | 165 cm |
|  | Weight |  | 15.1kg (18.2th percentile) | 46.5kg |
|  | Short stature (HP:0004322) | Frequent | Yes | Yes |
|  | Intrauterine growth retardation (HP:0001511) | Frequent | No | No |
| Head and neck | Downslanted palpebral fissures (HP:0000494) | Frequent | No | No |
|  | Micrognathia (HP:0000347) | Occasional | No | No |
|  | Cleft palate (HP:0000175) | Occasional | No | No |
|  | Epicanthus (HP:[0000286](https://hpo.jax.org/app/browse/term/HP:0000286)) |  | Yes | Yes |
|  | Upslanted palpebral fissure (HP:0000582) |  | No | Yes |
|  | Facial asymmetry (HP:0000324) |  | Yes (Mild) | Yes (Severe) |
| Ear | Hearing impairment (HP:0000365) | Occasional | Yes (Moderate sensorineural hearing impairment) (HP:0004322) | No |
|  | Anterior creases of earlobe (HP:0009908) | Occasional | No | No |
| Eye | Hypertelorism (HP:0004322) | Frequent | Yes | Yes |
|  | Chorioretinal coloboma (HP:0004322) | Frequent | Yes | No |
|  | Iris coloboma (HP:0000612) | Frequent | No | No |
|  | Microphthalmia (HP:0000568) | Occasional | No | No |
|  | Astigmatism (HP:0000483) |  | Yes | Yes |
|  | Abnormality of eye movement (HP:0000496) |  | Yes | Yes |
| Cardiovascular system | Abnormality of morphology | Frequent | Yes (Patent foramen ovale ) (HP:0001655) | No |
|  | Malformation of great vessels | Frequent | Yes (Pulmonary artery sling ) (HP:0004961) | No |
| Digestive System | Anal atresia (HP:0002023) | Very frequent | Yes | Yes |
|  | Rectoperineal fistula (HP:0004322) | Very frequent | Yes | No |
| Genitourinary system | Renal hypoplasia/aplasia (HP:0008678) | Frequent | No | No |
|  | Hydronephrosis (HP:0000126) | Frequent | No | No |
|  | Abnormal localization of kidney (HP:0000542) | Frequent | No | No |
|  | Abnormality of the genital system (HP:0000078) | Occasional | No | No |
| Skeletal system | Hip dysplasia (HP:0001385) | Frequent | Yes | No |
|  | Abnormality of the ribs (HP:0000772) | Frequent | No | No |
|  | Scoliosis (HP:0002650) |  | No | Yes |
| Limbs | Bilateral single transverse palmar creases (HP:0007598) |  | Yes | No |
| Musculature | Hypotonia (HP:0001252) | Frequent | Yes | No |
| Skin, Hair, and Nails | Preauricular pit (HP:0004467) | Very frequent | Yes | Yes |
|  | Preauricular skin tag (HP:0000384) | Very frequent | No | Yes |
| Nervous system | Global developmental delay (HP:0011342) | Frequent | Mild (PDMS-II and GDS) | N/A |
|  | Intellectual disability, mild (HP:0001256) | Frequent | N/A | Yes (MMSE=26) |
|  | Delayed myelination (HP:0012448) |  | Yes | No |
| Voice | Nasal speech (HP:0001611) |  | Yes | Yes |
| Results of genetic test | Karyotype analysis |  | 47,XX,+mar | 47,XY,+mar |
|  | CNV-seq |  | 22q11.1-q11.21(16,500,000-18,200,000)x4 | 22q11.1-q11.21(16,500,000-18,200,000)x4 |
| Diagnosis |  |  | cat eye syndrome | cat eye syndrome |

### N/A, not available; [PDMS-II, Peabody Developmental Motor Scale](https://www.so.com/link?m=bWifpuzEgcL/1vo1R18cOi4iKo9uf9dE5khTHqg0xyfWu3wsJX6eaNlWHsOOkuPS7OgTHDUg0/IhqtVoePOMCeeQYXRsi1YYvP/KEjPc7UyWwDpJg0gdQG0O3kjvPcL4PyhNNK06NLSghnQ6OAgKTTNiX0h9h2+6xEC3A7+ZVz3zn51YiM0b7Vg==)s II; GDS, Gesell Developmental Schedules; MMSE, Mini-mental State Examination; symptoms and data source: OMIM, HPO, Orphanet and 131 papers on CES published between 2001 and 2021;

**Supplementary Table 4.** Genes and Function.

| Gene | Name | Function | Related Condition |
| --- | --- | --- | --- |
| *XKR3*  (MIM *611674) | x kell blood group precursor-  related family, member3 | XKR3 is homologs of the kell blood group precursor XK, which is a membrane transporter and a component of the XK/kell complex of the Kell blood group system [3]. |  |
| *IL17RA*  (MIM *********605461) | interleukin 17 receptor A | IL17R is a receptor for IL17. IL17 induces nuclear factor kappa-B and expression of IL6, intercellular adhesion molecule-1, granulocyte macrophage colony-stimulating factor, and prostaglandin E2, as well as the maturation of CD34-positive hematopoietic precursors into neutrophils [4]. | Immunodeficiency 51 (IMD51) (MIM # [613953](https://www.omim.org/entry/613953)) |
| *CECR1*  (MIM *********607575)  or *ADA2* | cat eye syndrome chromosome region, candidate 1 or adenosine deaminase | ADA2 is an adenosine deaminase, and ADA-related growth factors, which are involved in tissue development. The various clinical manifestations of ADA2 deficiency, which include skin manifestations, vasculopathy, neuropathy and immunodeficiency [5]. | Sneddon syndrome (SNDNS) (MIM # [182410](https://www.omim.org/entry/182410))  Vasculitis,autoinflammation, immunodeficiency, and hematologic defects syndrome (VAIHS) (MIM # [615688](https://www.omim.org/entry/615688)) |
| *CECR**2*  (MIM *********607576) | cat eye syndrome chromosome region, candidate 2 | *CECR2* is play a role in neurulation during embryogenesis. Dysregulation of mesenchymal and ectodermal transcription factors and proteins triggered by *CECR2* mutation which its role appears to induce neurogenesis and inner ear development [6]. |  |
| *SLC25A18*  (MIM *********609303) | solute carrier family25 member 18 | The *SLC25A18* gene family encodes mitochondrial carriers that transport a variety of metabolites across the inner mitochondrial membrane [7], and the function of SLC25A18 primarily involves acting as a regulator of catalyzes the unidirectional transport of glutamate. |  |
| *ATP6V1E1*  (MIM ********* 609303) | ATPase V1 subunit E, isoform 1 | ATPase is responsible for the acidification of endosomes, lysosomes, and other intracellular organelles. It is also involved in hydrogen ion transport across the plasma membrane into the extracellular space [8]. | Cutis laxa, autosomal recessive, type II C (ARCL2C) (MIM #617402) |
| *BID*  (MIM ********* 601917) | BH3-interacting domain death agonist | *BID* encodes a cell death agonist and regulates apoptosis, and this gene play a role in inducing premature cell death could influence organ development or overall growth [9]. |  |
| *MICAL3*  (MIM ********* 608882) | microtubule-associated monooxygenase, calponin and limdomains-containing, 3 | *MICAL3*, a plexin signaling molecule, expressed in motor neurons [10], maybe directly associated with the impaired ocular movement. |  |
| *MIR648*  (MIM ********* 608882) | micro RNA 648 | Micro RNA are small noncoding RNAs that regulate gene expression primarily by binding to the 3-prime UTRs of MICAL3 pre-mRNA and promoting either mRNA degradation or translational inhibition [11]. |  |
| *PE*X26  (MIM ********* 115470) | peroxisome biogenesis factor 26 | *PEX26* response for encoding a member of the AAA protein family, a large group of ATPases associated with many cellular activities [12]. | Peroxisome biogenesis disorder 7A (Zellweger) (PBD7A) (MIM #[614872](https://www.omim.org/entry/614872))  Peroxisome biogenesis disorder (PBD7B) (MIM #[614873](https://www.omim.org/entry/614873)) |
| *TUBA8*  (MIM * 115470) | tubulin , alpha-8 | *TUBA8* is an evolutionary outlier within the mammalian a-tubulin family, it is found wide expression in developing neural structures and involved in optic nerve hypoplasia [13]. | Macrothrombocytopenia, isolated, 2, autosomal dominant (MACTHC2) (MIM #[619840](https://www.omim.org/entry/619840)) |


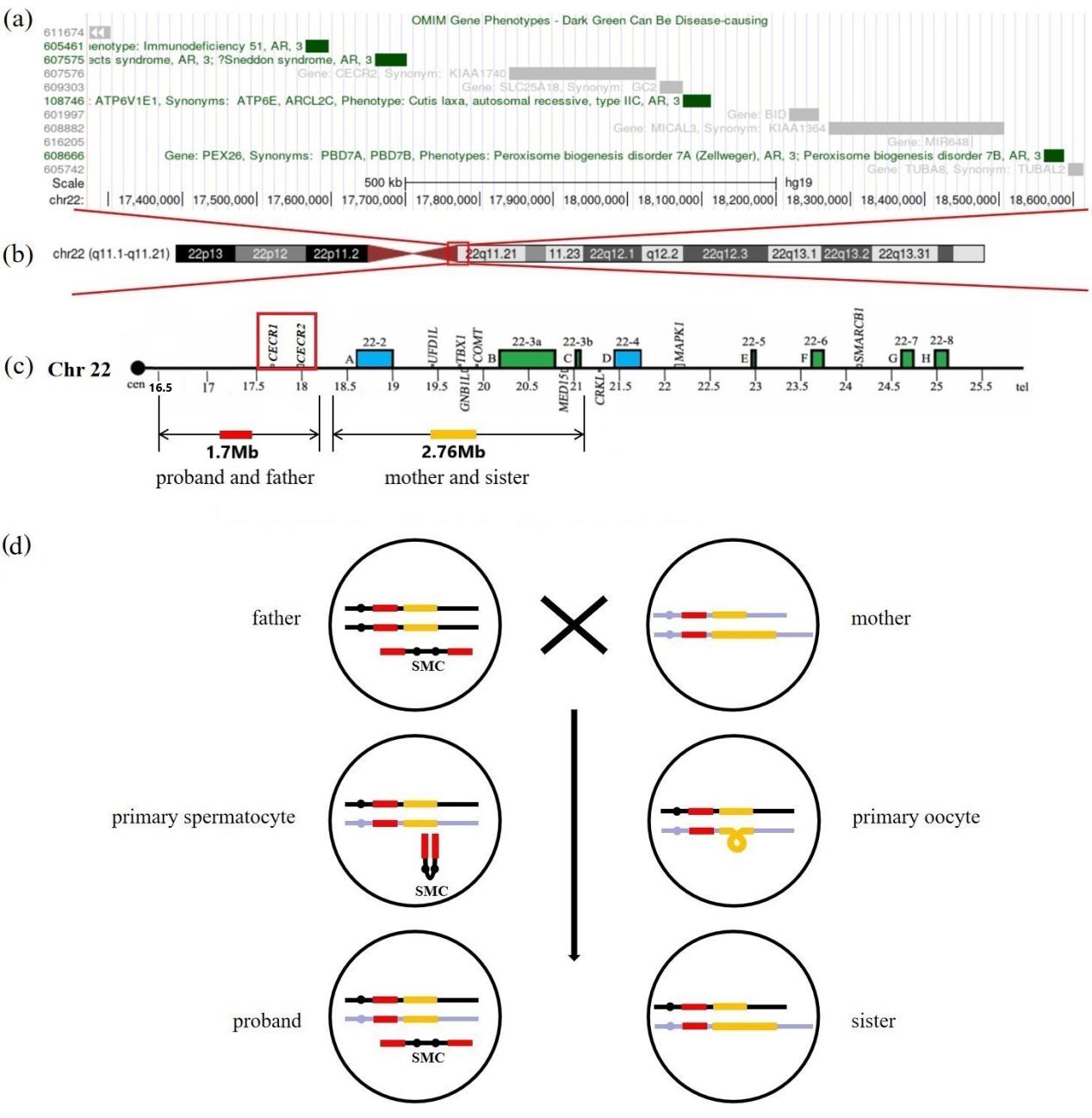


**Supplementary Fig. 1** The corresponding position of duplication on the chromosome and transmission. (a) 22q11.1-q11.21 (chr22:16,500,000-18,200,000, hg38) contains 11 OMIM morbid genes. (b) The standard stripes of chromosome 22 and location of duplication. (c) 22q11 region harbors eight LCRs termed LCR22A-LCR22H and the 1.7Mb duplication harbors *CECR1* and *CECR2* gene. (d) The duplication transmission.

**References:**

[1]. Scott A B,Kraft S P, Botulinum toxin injection in the management of lateral rectus paresis. Ophthalmology, 1985, 92: 676-683.

[2]. Farid Mohamed Fathy, Elbarky Ahmed Mohammed, Saeed Ahmed Mohamed, Superior rectus and lateral rectus muscle union surgery in the treatment of myopic strabismus fixus: three sutures versus a single suture. J AAPOS, 2016, 20: 100-105.

[3]. Calenda Giulia, Peng Jianbin, Redman Colvin M et al. Identification of two new members, XPLAC and XTES, of the XK family. Gene, 2006, 370: 6-16.

[4]. Lopez Kostka Susanna, Dinges Stephanie, Griewank Klaus et al. IL-17 promotes progression of cutaneous leishmaniasis in susceptible mice. J Immunol, 2009, 182: 3039-3046.

[5]. Lee Pui Y, Vasculopathy, Immunodeficiency, and Bone Marrow Failure: The Intriguing Syndrome Caused by Deficiency of Adenosine Deaminase 2. Front Pediatr, 2018, 6: 282.

[6]. Fabiola Quintero-Rivera, Julian A Martinez-Agosto, Hemifacial microsomia in cat-eye syndrome: 22q11.1-q11.21 as candidate loci for facial symmetry. Am J Med Genet A, 2013, 1985-1991.

[7]. Palmieri Ferdinando, The mitochondrial transporter family (SLC25): physiological and pathological implications. Pflugers Arch, 2004, 447: 689-709.

[8]. Van Damme Tim, Gardeitchik Thatjana, Mohamed Miski et al. Mutations in ATP6V1E1 or ATP6V1A Cause Autosomal-Recessive Cutis Laxa. Am J Hum Genet, 2017, 100: 216-227.

[9]. Dinsmore Colin J, Soriano Philippe, MAPK and PI3K signaling: At the crossroads of neural crest development. Dev Biol, 2018,S79-S97.

[10]. Bron Romke, Vermeren Matthieu, Kokot Natalie et al. Boundary cap cells constrain spinal motor neuron somal migration at motor exit points by a semaphorin-plexin mechanism. Neural Dev, 2007, 2: 21.

[11]. Griffiths-Jones Sam, Grocock Russell J, van Dongen Stijn et al. miRBase: microRNA sequences, targets and gene nomenclature. Nucleic Acids Res, 2006, 34: D140-144.

[12]. Matsumoto Naomi, Tamura Shigehiko, Fujiki Yukio, The pathogenic peroxin PEX26 precruits the Pex1p-Pex6p AAA ATPase complexes to peroxisomes. Nat Cell Biol, 2003, 5: 454-460.

[13]. Abdollahi Mohammad R,Morrison Ewan, Sirey Tamara et al. Mutation of the variant alpha-tubulin TUBA8 results in polymicrogyria with optic nerve hypoplasia. Am J Hum Genet, 2009, 85: 737-744.
